# Complete genome sequence and comparative analysis of *Acetobacter pasteurianus* 386B, a strain well-adapted to the cocoa bean fermentation ecosystem

**DOI:** 10.1186/1471-2164-14-526

**Published:** 2013-08-01

**Authors:** Koen Illeghems, Luc De Vuyst, Stefan Weckx

**Affiliations:** 1 Research Group of Industrial Microbiology and Food Biotechnology (IMDO), Faculty of Sciences and Bio-engineering Sciences, Vrije Universiteit Brussel, Pleinlaan 2, Brussels B-1050, Belgium

**Keywords:** *Acetobacter pasteurianus*, Cocoa bean fermentation, 454 pyrosequencing

## Abstract

**Background:**

*Acetobacter pasteurianus* 386B, an acetic acid bacterium originating from a spontaneous cocoa bean heap fermentation, proved to be an ideal functional starter culture for coca bean fermentations. It is able to dominate the fermentation process, thereby resisting high acetic acid concentrations and temperatures. However, the molecular mechanisms underlying its metabolic capabilities and niche adaptations are unknown. In this study, whole-genome sequencing and comparative genome analysis was used to investigate this strain’s mechanisms to dominate the cocoa bean fermentation process.

**Results:**

The genome sequence of *A. pasteurianus* 386B is composed of a 2.8-Mb chromosome and seven plasmids. The annotation of 2875 protein-coding sequences revealed important characteristics, including several metabolic pathways, the occurrence of strain-specific genes such as an endopolygalacturonase, and the presence of mechanisms involved in tolerance towards various stress conditions. Furthermore, the low number of transposases in the genome and the absence of complete phage genomes indicate that this strain might be more genetically stable compared with other *A. pasteurianus* strains, which is an important advantage for the use of this strain as a functional starter culture. Comparative genome analysis with other members of the *Acetobacteraceae* confirmed the functional properties of *A. pasteurianus* 386B, such as its thermotolerant nature and unique genetic composition.

**Conclusions:**

Genome analysis of *A. pasteurianus* 386B provided detailed insights into the underlying mechanisms of its metabolic features, niche adaptations, and tolerance towards stress conditions. Combination of these data with previous experimental knowledge enabled an integrated, global overview of the functional characteristics of this strain. This knowledge will enable improved fermentation strategies and selection of appropriate acetic acid bacteria strains as functional starter culture for cocoa bean fermentation processes.

## Background

Acetic acid bacteria (AAB) are a group of microorganisms that belong to the family of the *Acetobacteraceae* of the Alpha-proteobacteria [1]. AAB can be found on (tropical) fruits and flowers [2-4], in fermented foods [1,3], and as members of the *Drosophila* gut [5]. Overall, AAB are of industrial interest because of their physiology, which is the case for acetic acid production out of ethanol during vinegar, kombucha, or cocoa bean fermentation [6-8] as well as for fine chemical productions such as those of ascorbic acid and cellulose [9,10]. Furthermore, AAB can occur as spoilage bacteria, as can be the case in beer, wine, and cider fermentations [1,3]. One of the key metabolic features of AAB is the conversion of ethanol into acetic acid by two sequential reactions catalyzed by membrane-bound alcohol dehydrogenase (ADH) and aldehyde dehydrogenase (ALDH) enzymes [11].

Currently, AAB include twelve genera, among which *Acetobacter*, *Gluconobacter*, and *Gluconacetobacter* are the most studied ones [3,8,12]. The genus *Acetobacter* is one of the most interesting from a biotechnological point of view [1,3], because of its ability to oxidize ethanol into acetate while tolerating high acetic acid concentrations in the environment [13]. Different species within the *Acetobacter* genus are distinguished, among which *Acetobacter pasteurianus, Acetobacter aceti* and *Acetobacter pomorum* are important in industrial vinegar production [3,14,15], *Acetobacter cerevisiae* is present in beer and on grapes [16,17], and *Acetobacter tropicalis* and *Acetobacter senegalensis* are involved in the cocoa bean fermentation process [18]. *Acetobacter* species are able to oxidize acetate completely (so-called overoxidation) and use ubiquinones of the Q-9 type, the latter being in contrast with species of the AAB genera *Gluconacetobacter* and *Gluconobacter* that contain mainly ubiquinones of the Q-10 type [19]. At present, *A. pasteurianus* IFO 3283 (originating from a fermentation) is the only member of the genus *Acetobacter* of which the genome has been sequenced completely, including six plasmids [20]. However, draft genomes are available for *A. pasteurianus* 3P3 (originating from submerged wine vinegar) [21], *A. pasteurianus* NBRC 101655 (Thai pineapple) [22], *A. pasteurianus* subsp. *pasteurianus* LMG 1262^T^ (Dutch beer, type strain) [21], *A. aceti* NBRC 14818 (ethanol-based vinegar) [23], *A. pomorum* DM001 (*Drosophila* gut) [5], and *A. tropicalis* NBRC 101654 (Thai fruit) [2,24]. In general, *Acetobacter* species possess relatively small genomes (approximately 3 Mb), including plasmids in particular cases [20,25-27].

*Acetobacter pasteurianus* strains are used for vinegar fermentations worldwide [28-30] and also occur in beer as spoilers [3]. Further, it has been shown that this species plays an essential role in the fermentation of cocoa pulp-bean mass, the first step in chocolate production [31-33]. Spontaneous cocoa bean fermentation is characterized by a succession of microbial activities carried out by yeasts (in particular *Hanseniaspora opuntiae/uvarum* and *Saccharomyces cerevisiae*), involved in depectinization and ethanol formation; lactic acid bacteria (LAB, in particular *Lactobacillus fermentum*), involved in citric acid and fructose conversion and lactic acid production; and AAB (in particular *A. pasteurianus*), involved in the oxidation of ethanol produced by the yeasts into acetic acid and further overoxidation of acetic acid and lactic acid produced by LAB into carbon dioxide and water [6,34].

*Acetobacter pasteurianus* 386B originates from a spontaneous cocoa bean heap fermentation carried out in Ghana and has been characterized as an ethanol-oxidizing, lactic acid-oxidizing, and acetic acid-producing strain [18,35]. Furthermore, *A. pasteurianus* 386B is a thermotolerant strain with high resistance to ethanol and acetic acid [36,37]. These functional properties make it an ideal starter culture strain for cocoa bean fermentations [38]. In this study, we present the complete genome sequence and analysis of *A. pasteurianus* 386B to obtain insights into the genomic features of this interesting starter culture strain [37,38]. A better understanding of the molecular mechanisms underlying its metabolic capabilities will lead to detailed insights into the mechanisms of niche adaptation of this strain. Furthermore, comparison of *A. pasteurianus* 386B with other sequenced members of the *Acetobacteraceae* will address the unique functional properties of this strain as well as the common characteristics of the *Acetobacteraceae*.

## Results and discussion

### 454 Pyrosequencing and sequence annotation

**Figure 1:**
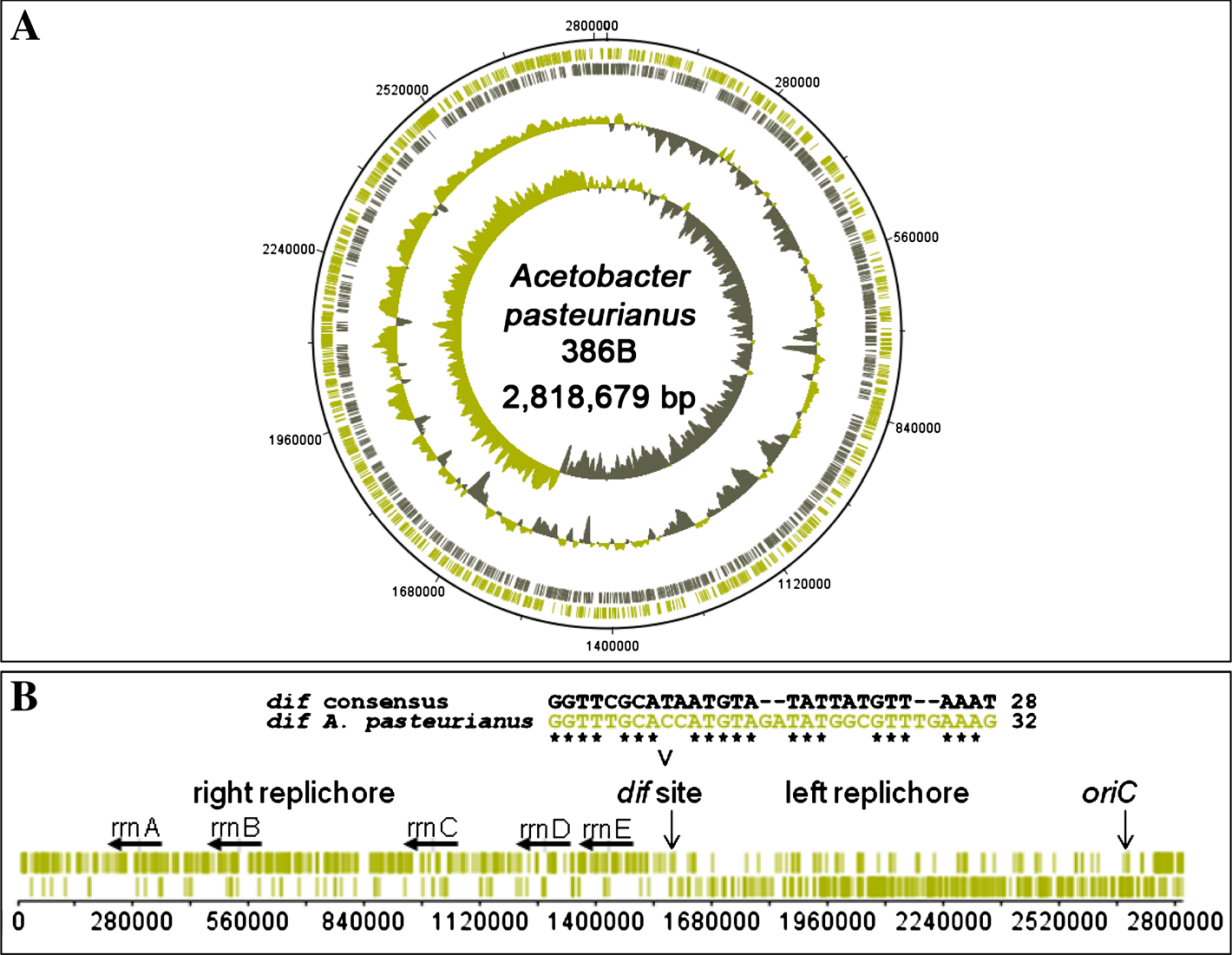
**Features of the *Acetobacter pasteurianus *386B chromosome. (A)** Circular representation of the annotated chromosome of *A. pasteurianus* 386B. The circles represent (from the outside to the inside): circle 1, DNA base position (bp); circle 2, protein-coding regions transcribed clockwise; circle 3, protein-coding regions transcribed anti-clockwise; circle 4, G+C content plotted using a 10-kb window in steps of 200 bp; circle 5, GC skew plotted using a 10-kb window in steps of 200 bp. **(B)** Linear representation of the *A. pasteurianus* 386B chromosome. The occurrence of the architecture imparting sequences (AIMS) is shown (in green) on both strands of the chromosome. These AIMS consist of the octamers [A|C|G]GGGCAGG, GGGCAGG[G|T], GAGCAGGG, GG[T|C]GAGGG, and GGG[C|A]AGGG. The origin of chromosomal replication (*oriC*) as well as the replication termination (*dif*) site are marked with a vertical arrow. The location of five rRNA operons (*rrnA*-*rrnE*) on the leading strand of the *A. pasteurianus* chromosome is indicated with horizontal black arrows.

The 454-pyrosequencing run of the *A. pasteurianus* 386B genomic DNA yielded 217,169 reads with a total number of 72,387,894 bp that were assembled into 10 scaffolds, consisting of 118 large (> 500 nucleotides) contigs and 25 small (100–500 nucleotides) contigs. Computational analysis of the sequence assembly indicated the presence of seven plasmids. The gaps in the chromosome and plasmids were closed by PCR assays followed by sequencing of the corresponding amplicons, resulting in a final assembly of the circular chromosome with a size of 2,818,679 bp and the seven plasmids ranging in size from 3,851 bp to 194,780 bp (Table 1; Figure 1A). Gene finding and annotation of the *A. pasteurianus* 386B genome with the GenDB software resulted in 2,595 and 280 protein-coding sequences (CDS) for the chromosome and plasmids, respectively. Furthermore, five ribosomal RNA (*rrn*) operons were detected and 57 tRNA genes were predicted. Clustered regularly interspaced short palindromic repeats (CRISPRs) were not found. Relevant features deduced from the genome sequence of *A. pasteurianus* 386B are summarized in Table 2.

**Table 1:** **General features of the *Acetobacter pasteurianus *386B chromosome and plasmids**

**Name abbreviation**	**EMBL accession no.**	**Contigs**	**Size (bp)**	**GC (%)**
APA386B (chromosome)	HF677570	78	2818679	52.91
APA386B_1P (plasmid)	HF677571	45	194780	52.01
APA386B_2P (plasmid)	HF677572	6	18169	55.57
APA386B_3P (plasmid)	HF677573	15	15601	55.47
APA386B_4P (plasmid)	HF677574	5	11105	52.28
APA386B_5P (plasmid)	HF677575	1	9914	50.75
APA386B_6P (plasmid)	HF677576	1	6,548	51.79
APA386B_7P (plasmid)	HF677577	2	3851	47.03

**Table 2:** **Features of the *Acetobacter pasteurianus *386B genome**

**Feature**	**Chromosome**	**Plasmid 1**	**Plasmid 2**	**Plasmid 3**	**Plasmid 4**	**Plasmid 5**	**Plasmid 6**	**Plasmid 7**
Total size (bp)	2818679	194780	18169	15601	11105	9914	6548	3851
G+C content (%)	52.91	52.01	55.57	55.47	52.28	50.75	51.79	47.03
No. of protein-coding genes	2595	220	20	11	9	11	5	4
Coding density (%)	89.7	82.4	72.6	61.8	73.8	58.6	34.8	90.2
Average gene length (bp)	975	729	660	876	911	528	456	869
No. of rRNA operons	5 × 16S-23S-5S	0	0	0	0	0	0	0
No. of tRNAs	57	0	0	0	0	0	0	0
No. of transposases	26	18	4	0	0	1	0	1

### General architecture of the *A. pasteurianus* 386B genome

A plot of the calculated G/C skew [(G–C)/(G + C)] indicated a bidirectional replication mechanism of the chromosome (Figure 1A), which was confirmed by the biased distribution of architecture imparting sequences (AIMS) on the leading and lagging strands [39], dividing the chromosome of *A. pasteurianus* 386B into two replichores of similar sizes (Figure 1B). This enabled the prediction of the origin of chromosomal replication (*oriC*), located near the *dnaA*-coding region (APA386B_66), as well as a replication termination (*dif*) region at position 1,590,515 on the chromosomal map [40]. The sequence of the 32-bp *dif* region was aligned with the consensus sequence of Gamma-proteobacterial *dif* sites [41]. The *dif* region, positioned opposite of the *oriC*, is involved in replication termination and defines the leading and lagging strands during replication, together with *oriC*[39]*.* The occurence of the G/C skew, replichores, and biased distribution of AIMS supports the accuracy of the sequence assembly, as they represent a common general architecture of a genome.

### Phylogenetic analysis and comparative genomics

**Figure 2:**
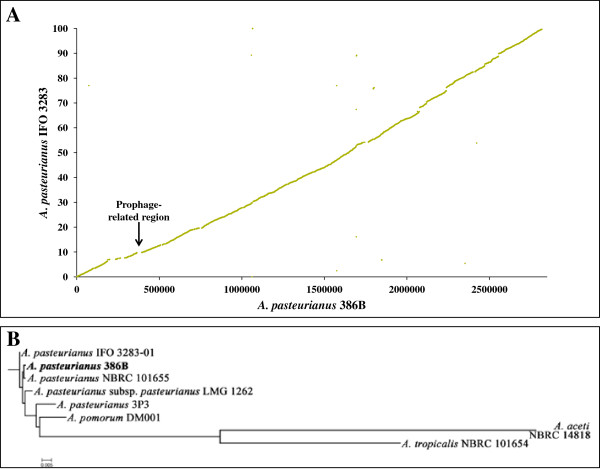
**Comparative analysis of the *Acetobacter pasteurianus *386B genome. (A)** Synteny plot between the chromosomes of *A. pasteurianus* 386B and *A. pasteurianus* IFO 3283. Each dot represents a predicted *A. pasteurianus* 386B protein having an ortholog in the *A. pasteurianus* IFO 3283 chromosome, with coordinates corresponding to the position of the respective coding region in each genome. The position of the putative prophage is marked with a vertical black arrow. **(B)** Phylogenetic tree based on all core genes of the strains included. Multiple sequence alignments of concatenated core gene sequences were calculated within EDGAR. Plasmid sequences were included.

Synteny analysis revealed a highly conserved order of orthologous genes between the genome sequences of *A. pasteurianus* 386B and *A. pasteurianus* IFO 3283 (Figure 2A). The chromosomal synteny was interrupted due to a few transposases/integrases and the presence of genes related to a prophage (Figure 2A). This prophage genomic segment had a size of about 28.8 kb and comprised 61 genes (APA386B_370 - APA386B_430). Thirteen, six, and six of these genes had homologues in the genomes of *A. pasteurianus* IFO 3283, *Gluconacetobacter diazotrophicus* Pal5, and *Gluconobacter oxydans* 621H, respectively (Additional file 1). The prophage-related region did not contain virulence-associated genes or genes coding for known proteins. However, the region contained an integrase (APA386B_430), a phage terminase (APA386B_403), and a portal protein (APA386B_401), amongst other prophage-related proteins. No head maturation protease, coat protein, or tail measure protein were retrieved, indicating that the prophage is defective [42]. Surprisingly, the genome sequence of *A. pasteurianus* 386B contained only 50 transposases (including plasmids; Additional file 1), none of which was of the IS1380-type, an insertion sequence abundant in multiple *A. pasteurianus* strains [20,43], indicating that the 386B strain is genetically more stable than currently known strains of *A. pasteurianus*[20]. In contrast, the genome sequence of *A. pasteurianus* IFO 3283 contains 280 transposons (75 transposases of the IS1380-type), which are involved in the truncation of 32 genes [20]. As these genes, such as a D-galactonate transporter (APA386B_203), an oxalyl-CoA decarboxylase (APA386B_504), a membrane-bound transporter (APA386B_1075), an acetyl-CoA:propanoate CoA-transferase (APA386B_2066), and an oxidoreductase (APA386B_1P21) were not truncated in *A. pasteurianus* 386B (Additional file 2), they may contribute to specific metabolic features of this strain. For instance, the presence of a functional acetyl-CoA:propanoate CoA-transferase may have implications on metabolic pathways involving acetate, an important substrate in the cocoa bean fermentation.

**Figure 3:**
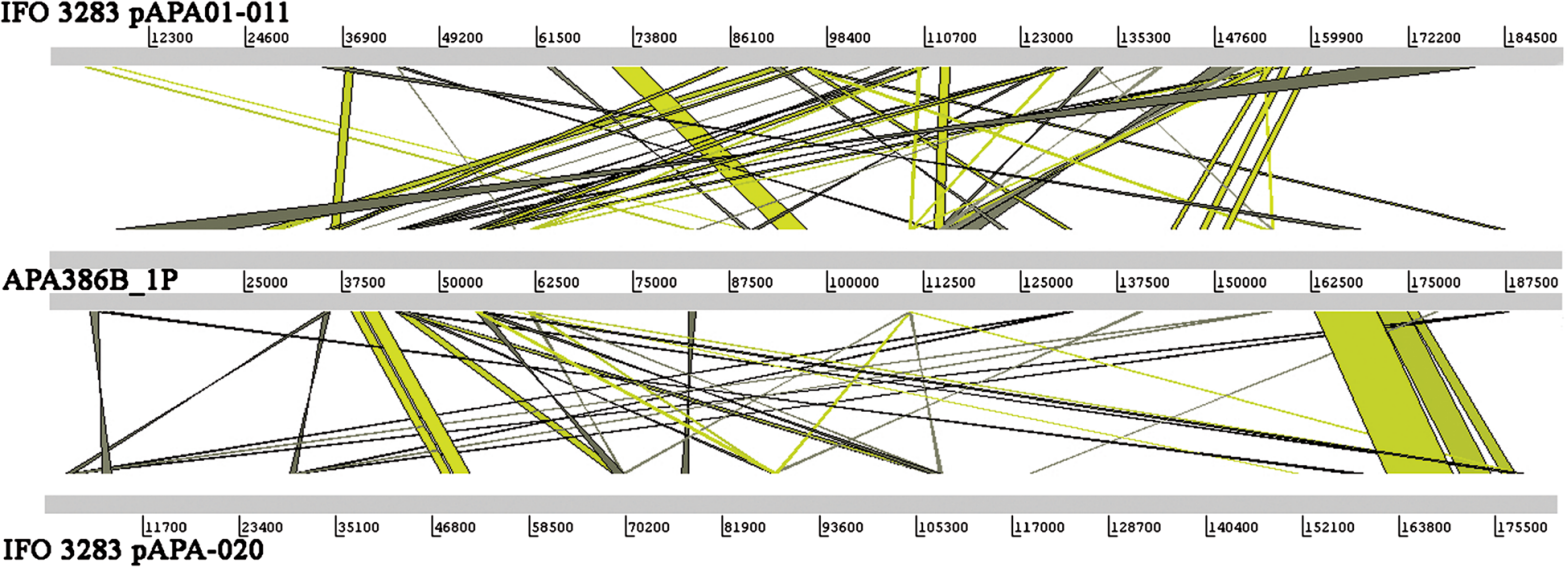
**Comparison of the large plasmids of *Acetobacter pasteurianus *386B and *A. pasteurianus *IFO 3283.** The ACT alignment is based on a BLASTN comparison using default settings. Green lines indicate that the aligned regions have the same orientation, whereas grey lines indicate that the aligned regions are inversely oriented.

A comparison of all seven plasmids of *A. pasteurianus* 386B with all six plasmids of *A. pasteurianus* IFO 3283 by means of the ACT and EDGAR software tools demonstrated that both strains did not share the same plasmids, as only very few homologous regions were retrieved (data not shown). For instance, pAPA01-011 and pAPA01-020, the largest plasmids of *A. pasteurianus* IFO 3283, shared only 44 and 16 CDS with APA386B_1P, the largest plasmid of *A. pasteurianus* 386B, respectively (Figure 3). Furthermore, APA386B_1P contained 165 unique genes, not shared with any of the two largest plasmids of *A. pasteurianus* IFO 3283.

Comparative analysis of the five available genome sequences of strains of the species *A. pasteurianus* revealed that there were 2,019 shared orthologous proteins, representing 68% of the predicted proteins from *A. pasteurianus* 386B. This may correspond with the core genome of the species *Acetobacter pasteurianus*. Furthermore, *A. pasteurianus* 386B contained 122 strain-specific genes, of which 95 had no known function, which may be related with niche adaptations. The 27 unique genes with an assigned function (Additional file 3) may contribute to the performance of this strain as a starter culture in the cocoa bean fermentation process. For instance, the presence of an endopolygalacturonase in the genome sequence of *A. pasteurianus* 386B (APA386B_1663; Additional file 3) indicates a possible role of this strain in pectin breakdown, an important metabolic process in the beginning of cocoa bean fermentations [44,45]. This is the first report of an endopolygalacturonase gene in an *A. pasteurianus* strain. Indeed, the closest relative possessing such a gene is *A. tropicalis* NBRC 101654 [2]; *A. tropicalis* has been isolated from spontaneous cocoa bean fermentation processes as well [32]. Furthermore, a PCR assay indicated that this polygalacturonase gene was not widespread amongst *A. pasteurianus* strains isolated from spontaneous cocoa bean fermentations (Additional file 4). This suggests that expression of this gene might contribute to the capability of *A. pasteurianus* 386B to dominate cocoa bean fermentations.

Phylogenetic analysis of the available genomes of the genus *Acetobacter* showed that the different *Acetobacter pasteurianus* strains were clustered together (Figure 2B). Furthermore, *A. pasteurianus* 386B was most related to *A. pasteurianus* NBRC 101655, a thermotolerant strain [46,47]. Comparative analysis of *A. pasteurianus* 386B in relation to other members of the *Acetobacteraceae* family revealed that this strain had approximately 20% shared genes with members of the genus *Acidiphilium* (Table 3). Furthermore, 27.1% of the genes are in common with the human pathogen *Granulibacter bethesdensis* CGDNIH1, whereas members of the genera *Gluconobacter* and *Gluconacetobacter* were more closely related to *A. pasteurianus* 386B, namely 35.6 and 34.5-39.1% shared genes, respectively. The finding that *A. pasteurianus* 386B had more genes in common with *Gluconacetobacter* species than with *G. oxydans* 621H was not in accordance with the phylogenetic relationship based on their complete 16S rRNA gene sequence [48]. This indicates that *Gluconacetobacter* species are more closely related to *Acetobacter* species than *Gluconobacter* species are.

**Table 3:** **Comparative analysis of *Acetobacter pasteurianus *386B and members of the *Acetobacteraceae *family**

**Acetic acid bacterium strain**	**Number of shared genes**	**Percentage of shared genes**
*Acetobacter pasteurianus* NBRC 101655	2492	76.6
*Acetobacter pasteurianus* IFO 3283	2344	69.5
*Acetobacter pasteurianus* LMG 1262^T^	2290	67.6
*Acetobacter pasteurianus* 3P3	2252	66.1
*Acetobacter pomorum* DM001	2067	64.7
*Acetobacter tropicalis* NBRC 101654	1980	44.8
*Acetobacter aceti* NBRC 14818	1657	31.7
*Gluconacetobacter diazotrophicus* Pal5	1603	34.5
*Gluconacetobacter medellinensis* NBRC 3288	1576	39.1
*Gluconobacter oxydans* 621H	1383	35.6
*Granulibacter bethesdensis* CGDNIH1	1128	27.1
*Acidiphilium multivorum* AIU301	1037	19.8
*Acidiphilium cryptum* JF-5	1023	20.9

Number of shared protein-coding sequences between *A. pasteurianus* 386B and members of the *Acetobacteraceae* family for which a complete genome sequence is available. The percentage of shared genes depicts the number of shared genes between two members in relation to the total amount of genes of both members.

### Intracellular metabolism of sugars and sugar derivatives

**Figure 4:**
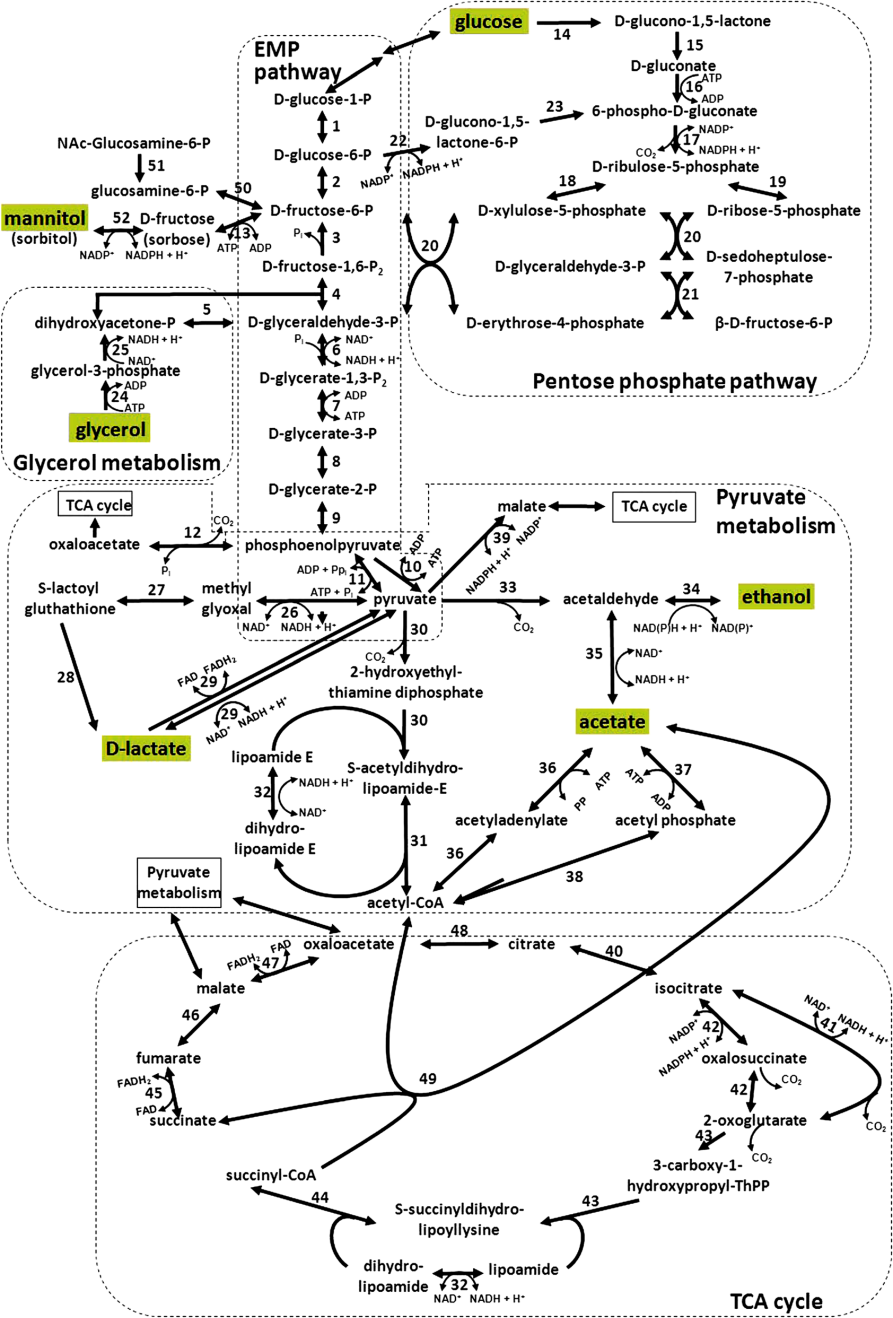
**Central metabolic pathways of *Acetobacter pasteurianus *386B.** (1) Phosphoglucomutase (APA386B_513); (2) glucose-6-phosphate isomerase (APA386B_1153); (3) fructose-1,6-biphosphatase I (APA386B_993); (4) fructose-biphosphate aldolase, class I (APA386B_1752); (5) triose-phosphate isomerase (APA386B_2746); (6) glyceraldehyde-3-phosphate dehydrogenase (APA386B_1520); (7) phosphoglycerate kinase (APA386B_1519); (8) phosphoglycerate mutase (APA386B_1895 and APA386B_2495); (9) enolase (APA386B_2618); (10) pyruvate kinase (APA386B_2108); (11) pyruvate-phosphate dikinase (APA386B_814); (12) phosphoenolpyruvate carboxylase (APA386B_587); (13) fructokinase (APA386B_356); (14) PQQ-dependent glucose dehydrogenase (APA386B_2133 - APA386B_2134); (15) gluconolactonase (APA386B_2110); (16) gluconate kinase (APA386B_1158); (17) phosphogluconate dehydrogenase (APA386B_1154); (18) ribulose-phosphate 3-epimerase (APA386B_2378); (19) ribose-5-phosphate isomerase A (APA386B_1157); (20) transketolase (APA386B_1152 and APA386B_1521); (21) transaldolase (APA386B_1153) (22) glucose-6-phosphate dehydrogenase (APA386B_729); (23) 6-phosphogluconolactonase (APA386B_1156); (24) glycerol kinase (Apa396B_92); (25) glycerol-3-phosphate dehydrogenase (APA386B_1931 and APA386B_94); (26) pyruvaldehyde oxidoreductase (APA386B_1946); (27) lactoylgluthathione lyase (APA386B_2164 and APA386B_1276); (28) hydroxyacylglutathione hydrolase (APA386B_2280); (29) lactate dehydrogenase (APA386B_910 and APA386B_1053); (30) pyruvate dehydrogenase E1 (APA386B_2083 - APA386B_2084 and APA386B_2737 - APA386B_2738); (31) pyruvate dehydrogenase E2 (APA386B_2085 and APA386B_2736); (32) dihydrolipoamide dehydrogenase (APA386B_2735 and APA386B_2271); (33) pyruvate decarboxylase (APA386B_1186); (34) alcohol dehydrogenase (APA386B_1507, APA386B_2362, and APA386B_496); (35) aldehyde dehydrogenase (APA386B_909); (36) acetyl-CoA synthetase (APA386B_1843 and APA386B_2214); (37) acetate kinase (APA386B_335); (38) phosphate acetyltransferase (APA386B_336); (39) malate dehydrogenase (APA386B_1600); (40) aconitate hydratase 1 (APA386B_1323); (41) NAD^+^-dependent isocitrate dehydrogenase (APA386B_2558); (42) NADP^+^-dependent isocitrate dehydrogenase (APA386B_2121); (43) 2-oxoglutarate dehydrogenase E1 (APA386B_2269); (44) 2-oxoglutarate dehydrogenase E2 (APA386B_2270); (45) fumarate reductase/succinate dehydrogenase (APA386B_1513 - APA386B_1516); (46) fumarate hydratase (APA386B_321 and APA386B_1305); (47) malate:quinone oxidoreductase (APA386B_2675); (48) citrate synthase (APA386B_2584); (49) succinyl-CoA:acetate CoA transferase (APA386B_2589); (50) glucosamine-6-phosphate isomerase (APA386B_546); (51) N-acetylglucosamine-6-phosphate deacetylase (APA386B_1531); (52) polyol oxidoreductase (APA386B_2545).

*Acetobacter pasteurianus* 386B possessed all genes encoding the enzymes of the Embden-Meyerhof-Parnas (EMP) pathway, except for the phosphofructokinase-coding gene, indicating incomplete glycolysis. The absence of this gene has been reported before in *A. pasteurianus* IFO 3283, *G. diazotrophicus* Pal5, and *G. oxydans* 621H [20,25,26]. However, all genes encoding the enzymes of the pentose-phosphate pathway (PPP) were found (Figure 4), suggesting that glucose is degraded via the PPP, as described previously for *A. pasteurianus* IFO 3283 and *A. aceti* NBRC 14818 [20,23]. Glucose, an important substrate in the cocoa bean fermentation process, could be taken up by sugar permeases (APA386B_1532 and APA386B_2419) or a sugar symporter (APA386B_1333). Fructose-6-phosphate could be formed from N-acetyl-glucosamine-6-phosphate as well as from mannitol via fructose by a polyol oxidoreductase (APA386B_2545; Figure 4, reaction 52). Fructose-6-phosphate could be further metabolized by the EMP pathway (Figure 4). Furthermore, the gene coding for glycerol kinase (*glpK*; Apa396B_92; Figure 4, reaction 24) was found, allowing the formation of dihydroxyacetone (DHA)-phosphate from glycerol via glycerol 3-phosphate, which could be further metabolized by the EMP pathway. DHA-phosphate could be formed by both FAD- and NAD-dependent glycerol 3-phosphate dehydrogenases (APA386B_1931 and APA386B_94; Figure 4, reaction 25). Next to this, the *A. pasteurianus* 386B genome contained genes coding for a glycerol uptake facilitator protein (APA386B_93), suggesting that this strain is able to take up glycerol from the environment to use it as an energy source, which might be present as a substrate during the cocoa bean fermentation process owing to yeast metabolism. DHA-phosphate may be channeled into the lower part of the EMP pathway. The *A. pasteurianus* 386B genome sequence provided further evidence that acetate is formed out of ethanol by soluble, NAD(P)^+^-dependent ADH (*adh*; APA386B_1507; Figure 4, reaction 34) and ALDH (APA386B_909; Figure 4, reaction 35) intracellularly, or out of lactate by means of a lactate dehydrogenase (*ldh*; APA386B_910; Figure 4, reaction 29) and pyruvate decarboxylase (*pdc*; APA386B_1186; Figure 4, reaction 33), as proposed before [49]; F. Moens, T. Lefeber, and L. De Vuyst, [unpublished observations]. Acetate, formed intracelluarly or available extracellularly, could be further (over)oxidized via acetyl-CoA into carbon dioxide and water by a modified tricarboxylic acid (TCA) cycle, as explained below. Genes encoding enzymes of the glyoxylate pathway were not present. During the course of all aforementioned reactions, NAD(P)H+H^+^ is produced by several dehydrogenases. Indeed, next to the annotated dehydrogenases, the *A. pasteurianus* 386B genome revealed several putative dehydrogenases/oxidoreductases with a currently unknown function (Additional file 5). These uncharacterized oxidoreductases included four aldehyde dehydrogenases, 15 short-chain dehydrogenases/reductases (involved in oxidation of alcohols to aldehydes), a zinc-binding dehydrogenase, and an oxidoreductase containing a NAD^+^-binding Rossmann-fold domain. Intracellular dehydrogenase activity is indispensable for the intermediary metabolism of AAB [25].

Genome analysis showed that *A. pasteurianus* 386B possessed genes encoding metabolic pathways involved in the *de novo* synthesis of all nucleotides, amino acids, phospholipids and many vitamins, such as biotin, folic acid, pantothenate, pyridoxine, riboflavin, and thiamine. Ammonia, involved in the activity of glutamate synthase (APA386B_893 - APA386B_894) and glutamine synthetase (APA386B_2129), could be taken up by a specific ammonia transporter (APA386B_239). Furthermore, the genome of *A. pasteurianus* 386B contained genes to synthesize and use trehalose, which can protect the cell from high osmolarity and/or can be used as an energy source in bacteria and yeast [50]. The pathway consisted of trehalose-6-phosphate synthase (*otsA*; APA386B_1724), trehalose-6-phosphate phosphatase (*otsB*; APA386B_1723), and trehalase (*treA*; APA386B_104). In addition, genes coding for the mechanosensitive channels MscL (APA386B_2572) and MscS (APA386B_1440) were present in the genome sequence of *A. pasteurianus* 386B, which generally play an important role in osmotolerance [51].

### Membrane-bound dehydrogenases and respiratory chain

**Figure 5:**
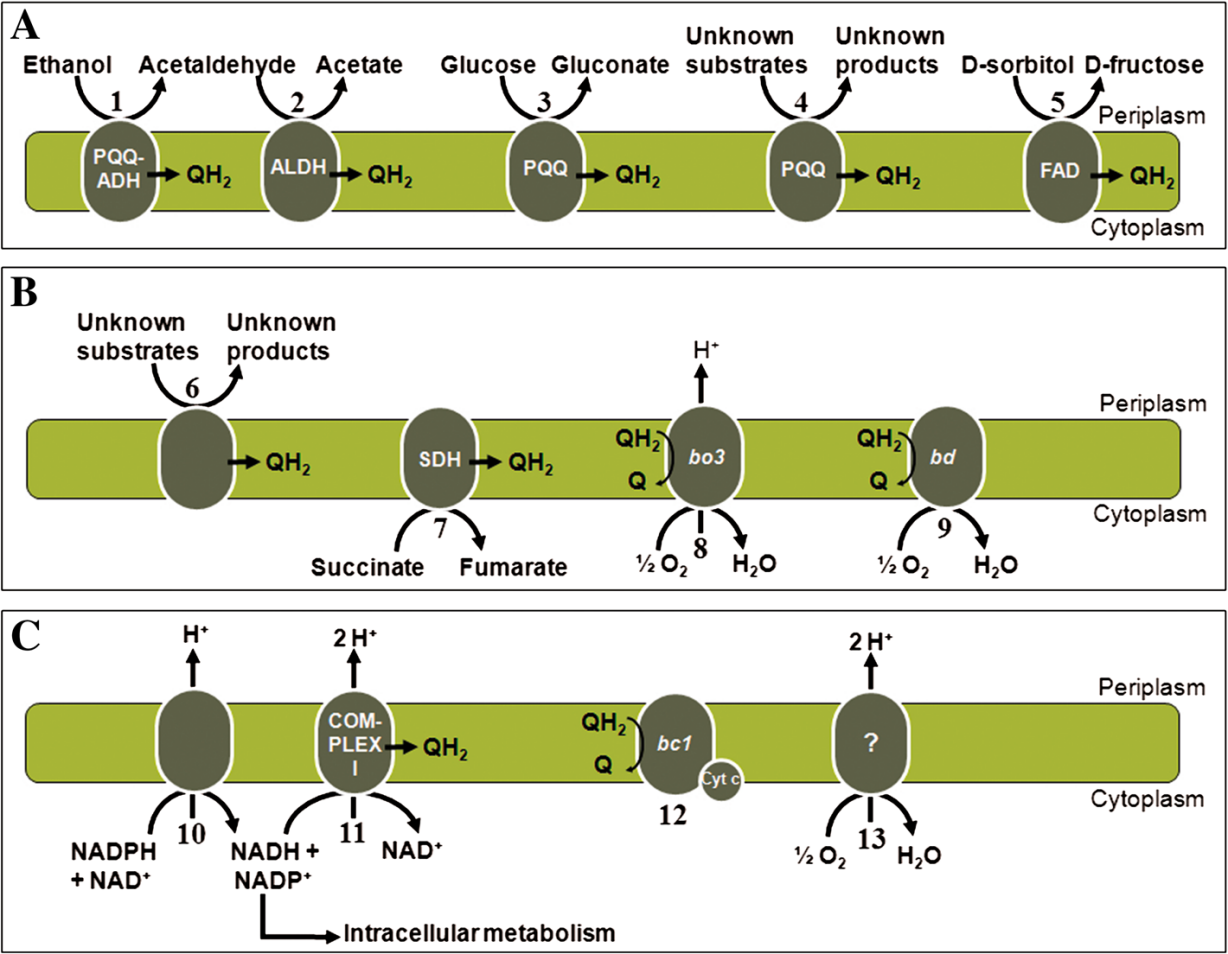
**Respiratory chain of *Acetobacter pasteurianus *386B. (A)** Membrane-bound PQQ- and FAD-dependent dehydrogenases: (1) PQQ-dependent alcohol dehydrogenase (APA386B_1574 - APA386B_1575); (2) membrane-bound acetaldehyde dehydrogenase (APA386B_2542 - APA386B_2544); (3) PQQ-dependent glucose dehydrogenase (APA386B_2133 - APA386B_2134); (4) uncharacterized PQQ-containing oxidoreductases [APA386B_1016 (transmembrane regions: 20–42, 47–64, 69–86, 101–120, 127–149) and APA386B_325 (transmembrane regions: 7–29, 33–55, 64–86, 90–107, 120–142)]; (5) FAD-dependent sorbitol dehydrogenase (APA386B_1096 - APA386B_1098). **(B)** Membrane-bound oxidoreductases and terminal oxidases**:** (6) uncharacterized oxidoreductases [APA386B_1815 (transmembrane region: 133–153), APA386B_1888 (transmembrane region: 307–329), APA386B_2052 (transmembrane region: 9–31), APA386B_767 (transmembrane region: 36–102), APA386B_1507 (transmembrane region: 168–188), and APA386B_2362 (transmembrane regions: 164–184, 222–242)]; (7) succinate dehydrogenase/fumarate reductase (APA386B_1513 - APA386B_1516); (8) cytochrome *bo*_*3*_ ubiquinol oxidase (APA386B_1578 - APA386B_1581); (9) cytochrome *bd* ubiquinol oxidase (APA386B_472 - APA386B_473 and APA386B_1010-APA386B_1011). **(C)** Respiratory chain core system: (10) proton-translocating transhydrogenase (APA386B_1508 - APA386B_1510); (11) proton-translocating NADH:ubiquinone oxidoreductase (complex I; APA386B_556 - APA386B_567 and APA386B_2247); (12) *bc*_*1*_ complex ubiquinol:cytochrome *c* oxidoreductase (complex III; APA386B_775 - APA386B_777); (13) incomplete cytochrome *c* oxidase (APA386B_609). Cyt c: cytochrome *c* (APA386B_367 and APA386B_906).

*Acetobacter pasteurianus* 386B possessed several membrane-bound dehydrogenases that channel electrons into the respiratory chain (Figure 5A). A first group of dehydrogenases depend on the cofactor pyrroloquinoline quinone (PQQ), among which PQQ-dependent alcohol dehydrogenase (PQQ-ADH) and PQQ-dependent glucose dehydrogenase, allowing the conversion of ethanol into acetaldehyde and glucose into gluconate, respectively, both substrates being available during cocoa bean fermentations. The genes coding for the three subunits of PQQ-ADH were not clustered together in the genome, as the gene encoding the smallest subunit (*adhS*; APA386B_2212) was separated from the other two genes (*adhAB*; APA386B_1574 - APA386B_1575). This gene organization in *A. pasteurianus* has been suggested before [52]. Further, the genome of *A. pasteurianus* 386B contained two uncharacterized, membrane-bound, PQQ-dependent oxidoreductases with five transmembrane helices (Additional file 5). Proteins for the synthesis of the cofactor PQQ are encoded by the *pqqABCDE* operon (APA386B_983 - APA386B_987). In contrast to other AAB, the genome of *A. pasteurianus* 386B did not possess the major polyol dehydrogenase (SldAB), an enzyme of industrial importance [53]. Indeed, SldAB is for instance able to oxidize D-sorbitol into L-sorbose (as part of the production of vitamin C that is used as food supplement and antioxidant), gluconate into 5-ketogluconate (as part of the production of tartaric acid that is used as antioxidant in the food industry), and glycerol into dihydroxyacetone (used in self-tanning creams) [8,25].

A second group of membrane-bound dehydrogenases contains flavines as cofactor. The genes coding for flavine adenine dinucleotide (FAD)-dependent sorbitol dehydrogenase (APA386B_1096 - APA386B_1098) were present in the *A. pasteurianus* 386B genome, which points to the ability of this strain to produce fructose from sorbitol. However, it has been shown previously that this dehydrogenase is also responsible for the conversion of mannitol, an important intermediate of the cocoa bean fermentation process, into fructose [54]. As the major polyol dehydrogenase was not present in this strain, it is likely that the FAD-dependent sorbitol dehydrogenase was responsible for the oxidation of mannitol into fructose, as experimentally shown in *A. pasteurianus* 386B (F. Moens, T. Lefeber, and L. De Vuyst, unpublished observations). In addition, *A. pasteurianus* 386B possessed six membrane-bound oxidoreductases with unknown function (Figure 5B; Additional file 5). These oxidoreductases are also present in the genomes of *A. pasteurianus* IFO 3283, *Ga. diazotrophicus* Pal5, and *G. oxydans* 621H (Additional file 6), and could be involved in the oxidation of a broad range of substrates, such as carbohydrates and polyols [25]. Genome analysis revealed that ubiquinol, generated by the aforementioned membrane-bound dehydrogenases, could be reoxidized by a cytochrome *bo3*-type ubiquinol oxidase (APA386B_1578 - APA386B_1581) and a cytochrome *bd*-type ubiquinol oxidase (cyanide-insensitive terminal oxidase), whereby the encoding genes of the latter were present twice in the genome sequence of *A. pasteurianus* 386B (*cydAB*; APA386B_472 - APA386B_473 and APA386B_1010 - APA386B_1011). Both terminal oxidases reduce oxygen to water when reoxidizing ubiquinol into ubiquinone (Figure 5B).

The genes coding for a proton-translocating nicotinamide nucleotide transhydrogenase were present in the genome of *A. pasteurianus* 386B (*pnt*; APA386B_1508 - APA386B_1510; Figure 5C). This enzyme might oxidize NADPH+H^+^ derived from the intermediary metabolism, thereby translocating protons across the cytoplasmic membrane. The NADH+H^+^ derived from transhydrogenase activity might subsequently be reoxidized by complex I of the respiratory chain, whereas the NADP^+^ derived from the transhydrogenase can be reduced by the NADP^+^-dependent ALDH [55]. Indeed, the genes coding for a complete proton-translocating respiratory chain complex I were retrieved (*nuoA*-*nuoN*; APA386B_556 - APA386B_567; Figure 5C). This complex I is absent in *G. oxydans*, which may be related to its lower growth yields [25]. Furthermore, a membrane-bound succinate dehydrogenase/fumarate reductase was found (*sdhABCD*; APA386B_1513 - APA386B_1516; Figure 5C), which is not only part of the respiratory chain, but also plays an important role in the TCA cycle, thereby enabling overoxidation of acetic acid [56]. Genes encoding both a *bc*_*1*_ complex (ubiquinol:cytochrome *c* oxidoreductase; *petABC*; APA386B_775 - APA386B_777) as well as cytochrome *c* (*cytC*; APA386B_367 and APA386B_906) were identified in the genome of *A. pasteurianus* 386B. Furthermore, genes for the transport of heme c and the maturation of cytochrome *c* were present (*ccmFGHI*; APA386B_2396 - APA386B_2400) [57]. However, it is unknown if this strain is able to reoxidize the reduced form of cytochrome *c,* as cytochrome *c* oxidase (complex IV) is probably inactive, because only subunit I (APA386B_609) was present in the genome.

### *In silico* analysis of mechanisms involved in acid tolerance

A first strategy of *A. pasteurianus* 386B to tolerate high levels of acetic acid may consist of a cytosolic acetate-assimilating detoxification pathway. This involves a conversion of acetate to acetyl-CoA, which is performed either by acetyl-CoA synthetase (*acn*; APA386B_2214 and APA386B_1843; Figure 4, reaction 36) or by acetate kinase (*ackA*; APA386B_335; Figure 4, reaction 37) and phosphate acetyltransferase (*pta*; APA386B_336; Figure 4, reaction 38). Both pathways were present in the *A. pasteurianus* 386B genome and are known to be upregulated when citrate oxidation takes place [58]. This suggests that the presence of two copies of the *acn* gene in this strain provides an advantage for efficient acetate assimilation. Alternatively, acetate can be converted into acetyl-CoA via a modified TCA cycle [59]. Indeed, all genes encoding the enzymes of the TCA cycle were retrieved, except for succinyl-CoA synthetase. This function is bypassed by succinyl-CoA: acetate CoA transferase (SCACT, *aarC*; APA386B_2589; Figure 4, reaction 50). Similarly, the gene for malate dehydrogenase was not found, but oxidation of malate into oxaloacetate can be catalyzed by malate:quinone oxidoreductase (*mqo*; APA386B_2675; Figure 4, reaction 48) [59]. A second mechanism in acid tolerance probably involves the presence of an acetic acid resistance ABC transporter (*aatA*; APA386B_103), an efflux pump in the cytoplasmic membrane capable of exporting acetic acid [60]. Thirdly, *A. pasteurianus* 386B contained the gene cluster involved in pellicle polysaccharide formation (*polABCDE*; APA386B_1394 - APA386B_1398), preventing the diffusion of acetic acid into the cytoplasm [46,61,62]. Fourthly, the genes coding for urease (*ureDABCEFG*; APA386B_1179 - APA386B_1184), an urea transporter (*urtABCDE*; APA386B_1640 - APA386B_1644), an allophanate hydrolase (APA386B_936 - APA386B_937), and an urea carboxylase (APA386B_218) were present, indicating the ability to transport urea and convert it into ammonia, which may contribute to the survival of *A. pasteurianus* 386B in acidic environments, such as the cocoa pulp-bean mass (pH 3.5 – 4.5). The human pathogenic *Gr. bethesdensis* CGDNIH1 contains this mechanism too, although it may not be widespread among AAB strains, as it is absent in *G. oxydans* 621H and *Ga. diazotrophicus* Pal5 [25,26,63]. Lastly, genome analysis of *A. pasteurianus* 386B revealed the presence of genes coding for cytoplasmic components that are adapted to intracellular acidification. This is the case for *N*^5^-carboxyaminoimidazole ribonucleotide (*N*^5^-CAIR) mutase (*purE*; APA386B_2565), a protein involved in purine biosynthesis. Indeed, *N*^5^-CAIR mutase of *A. pasteurianus* 386B is 99% identical to its orthologue in *A. aceti* 1023, the latter strain being adapted to an acid cytosol [64]. Similarly, alanine racemase (*alr*; APA386B_1310), a protein involved in peptidoglycan biosynthesis, is 92% identical to the *A. aceti* 1023 orthologue, a protein known to function at low pH [65]. In addition, the sequence similarity of both *N*^5^-CAIR mutase and alanine racemase between *A. pasteurianus* 386B and *A. aceti* 1023 was higher than between *A. pasteurianus* 386B and any other sequenced AAB strain (data not shown), indicating that the presence of acid-stable proteins is not widespread.

### *In silico* analysis of mechanisms involved in thermotolerance

As described above, genome-wide phylogenetic analysis of *A. pasteurianus* 386B revealed that this strain is most related to the thermotolerant strain *A. pasteurianus* NBRC 101655. In addition, adaptive mutation resulted in 14 validated mutations, involved in improved thermotolerance of this strain [66]. Five of these regions were also modified in *A. pasteurianus* 386B and not in *A. pasteurianus* NBRC 101655 (Additional file 7). For example, one of the two genes coding for cytochrome *c* (APA386B_906), contained three synonymous mutations and one nonsynonymous mutation. Furthermore, the genes necessary for growth at high temperatures (42°C) of the thermotolerant strain *A. tropicalis* NBRC 101654 have been identified recently [4]. Although this strain belongs to a different species than *A. pasteurianus* 386B, all genes of *A. tropicalis* NBRC 101654 necessary for growth at high temperatures were found in the genome sequence of *A. pasteurianus* 386B as well (Additional file 7). This indicates that the latter strain, when thriving in the high-temperature cocoa pulp-bean mass, may use the same mechanisms towards heat stress as *A. tropicalis* NBRC 101654.

## Conclusions

The complete genome sequence of *A. pasteurianus* 386B, a strain originating from a spontaneous cocoa bean heap fermentation in Ghana, was determined, annotated, and described in this study. The global overview of all genes and pathways obtained provided comprehensive insights into the metabolic features regarding important substrates (such as ethanol, glucose, acetate, lactate, and glycerol) and stresses (such as acidic and heat stress) during the cocoa bean fermentation process.

Comparative genome analysis provided information regarding niche adaptations of this strain. For example, the presence of a gene coding for an endopolygalacturonase was discovered. This enzyme is involved in the breakdown of pectin, a compound responsible for the viscosity of the cocoa pulp-bean mass. Although depectinization is mainly important in the beginning (anaerobic yeast activity phase) of the cocoa bean fermentation process, the activity of the pectinolytic enzymes allows air to enter the cocoa pulp-bean mass, which promotes the growth of obligate aerobic AAB. Therefore, the presence of this gene could be an important prerequisite for survival and performance of AAB during cocoa bean fermentations. Furthermore, the comparative genome analysis revealed that the genome of *A. pasteurianus* 386B contained a low number of transposases, resulting in the absence of truncated genes, which might be important for expression under cocoa bean fermentation conditions.

Genome analysis unraveled various mechanisms of *A. pasteurianus* 386B to tolerate stress conditions occurring in a cocoa bean fermentation ecosystem. As also active prophages were absent in the genome sequence, these findings indicate that *A. pasteurianus* 386B is genetically more stable compared with other fully characterized AAB, contributing to the prerequisites of a starter culture strain.

All these findings support that this strain is a suitable functional starter culture for controlled cocoa bean fermentation processes. In addition, the results presented in this study will enable analysis of the transcriptome of *A. pasteurianus* 386B, which will provide insight into its metabolic activity. Finally, the characteristics of *A. pasteurianus* 386B revealed in this study are essential to generate further insights into the functional role of AAB in general, and *A. pasteurianus* in particular, during the cocoa bean fermentation process, which is of great importance to select an appropriate starter culture for homogeneous, fast, and successfully controlled fermentation processes.

## Methods

### Bacterial strain and growth conditions

*Acetobacter pasteurianus* 386B was originally isolated from a spontaneous cocoa bean heap fermentation carried out in Ghana [18]. The strain was stored at −80°C in mannitol-yeast extract-peptone (MYP) medium [2.5% D-mannitol, 0.5% yeast extract, and 0.3% bacteriological peptone (Oxoid, Basingstoke, Hampshire, United Kingdom), w/v], supplemented with 25% (v/v) glycerol as a cryoprotectant. To obtain cell pellets, *A. pasteurianus* 386B was propagated in MYP medium twice (aerobic incubation at 30°C overnight), followed by centrifugation (21,036 × *g*, 15 min, 4°C) of 2-ml cultures.

### DNA extraction and 454 pyrosequencing

Total genomic DNA was extracted from cell pellets using the High Pure PCR Template Preparation Kit (Roche Applied Science, Mannheim, Germany), followed by RNase treatment and purification using the High Pure PCR Product Purification Kit (Roche Applied Science), always according to the manufacturer’s instructions. To confirm the identity of the bacterial strain grown, a 16S rRNA gene-specific region was amplified based on the genomic DNA extracted, as described previously [67]. Amplicons were purified using the Wizard SV Gel and PCR Clean up system (Promega, Madison, WI, USA) and sequenced at a commercial facility using Sanger sequencing (VIB Genetic Service Facility, Antwerp, Belgium). The quality of the genomic DNA was assessed by gel electrophoresis; its quantity was estimated by a fluorescence-based method using the Quant-iT dsDNA Assay kit (Invitrogen, Carlsbad, CA, USA) and the DTX800 multimode detector (Beckman Coulter, Pasadena, CA, USA).

For genome sequencing, a total amount of 5 μg of genomic DNA was used for the construction of an 8-kb paired-end library with the GS FLX Titanium Library Paired-End Adaptors Kit and the GS FLX Titanium Rapid Library Prep Kit (Roche Applied Science) according to the manufacturer’s instructions. The optimal DNA copy per bead ratio was determined by an emulsion PCR titration using a GS FLX Titanium SV emPCR kit (Lib-L) (Roche Applied Science). The final emulsion PCR was performed using the GS FLX Titanium LV emPCR kit (Lib-L; Roche Applied Science). Pyrosequencing was performed on a Genome Sequencer GS FLX instrument using Titanium chemistry (Roche Applied Science) with the sample occupying one region of a four-region gasket. Library preparation and pyrosequencing were performed by the VIB Nucleomics Core Facility (Leuven, Belgium). Reads were assembled using the GS De Novo Assembler version 2.5.3 with default parameters.

### PCR-based gap closure

To close remaining gaps in the assembled genome sequence, PCR primers were designed based on contig ends using the Consed program [68] and synthesized in a commercial facility (Integrated DNA Technologies, Leuven, Belgium). PCR assays were performed using a DNA T3000 thermocycler (Biometra, Goettingen, Germany), containing 50 ng of genomic DNA, 100 μM of each dNTP (Sigma-Aldrich, St. Louis, MO, USA), 5 pmole of each primer, 5 μL of 10 × PCR reaction buffer (Fermentas, St. Leon-Rot, Germany), 1.875 U of *Pfu* DNA Polymerase (Fermentas), and sterile ultrapure water in a final volume of 50 μL. Following amplification, PCR product sizes were verified using a 1.0-% (w/v) agarose gel and the remaining reaction mixture was purified using the Wizard SV Gel and PCR Clean up system (Promega). Amplicons were sequenced in a commercial facility using Sanger sequencing technology (Macrogen Europe, Amsterdam, The Netherlands). All DNA sequences obtained were uploaded into the Consed program, manually inspected, and integrated into the genome assembly to generate the complete genome sequence of *A. pasteurianus* 386B. To facilitate gap closure and assembly validation, contigs were mapped to the *A. pasteurianus* IFO 3283 genome by means of the r2cat tool [20,69].

### Genome analysis and annotation

Automatic gene prediction and annotation of the assembled genome sequence were carried out using a local installation of the bacterial genome annotation system GenDB v2.2 [70]. A combined gene prediction strategy was applied by using GLIMMER 2.1 and the CRITICA program suite [71,72]. Putative ribosomal binding sites and tRNA genes were identified with the RBSfinder tool [73] and tRNAscan-SE [74]. The deduced proteins were functionally characterized by REGANOR [75] using automated searches in public databases, including SWISS-PROT and TrEMBL [76], Pfam [77], KEGG [78], and TIGRFAM [79]. Additionally, SignalP (detection of signal peptides) [80], helix-turn-helix (identification of helix-turn-helix DNA binding motifs) [81], and TMHMM (detection of transmembrane regions) [82] were applied. Each gene was functionally classified by assigning a Cluster of Orthologous groups (COG) number [83] and a Gene Ontology (GO) number [84]. The automated gene prediction and annotation was followed by manual curation of the data. To correct for over-annotation, short CDS without functional annotation, with low confidence scores inferred by the GenDB platform, and with overlaps with other CDS were eliminated from the final annotation. A genome plot of *A. pasteurianus* 386B was generated with the DNAPlotter tool [85]. The origin of chromosomal replication of *A. pasteurianus* 386B was predicted with the Ori-Finder tool [40]. CRISPRs were searched for with the CRISPRFinder tool [86].

### Phylogenetic analysis and comparative genomics

A phylogenetic analysis was performed using complete and draft genome sequences of members of the family *Acetobacteraceae*. Therefore, the annotated genome sequences of the finished genomes of *A. pasteurianus* IFO 3283 (including plasmids), *Acidiphilium multivorum* AIU301, *Acidiphilium cryptum* JF-5, *Ga. diazotrophicus* Pal5, *Ga. medellinensis* NBRC 3288 (formerly *Gluconacetobacter xylinus* NBRC 3288), and *G. oxydans* 621H were used [25-27,63,87]. Furthermore, the draft genome sequences (contigs and scaffolds) of *A. pasteurianus* subsp. *pasteurianus* LMG 1262^T^, *A. pasteurianus* NBRC 101655, *A. pomorum* DM001, *A. tropicalis* NBRC 101654, and *A. aceti* NBRC 14818 were included [2,5,20,22,23]. As no annotation of the draft genome sequence of *A. pasteurianus* 3P3 [21] was available, the draft genome was annotated using the GenDB platform as described above. The manually curated genome sequence of *A. pasteurianus* 386B, together with the plasmids identified, was incorporated as well. Comparative analysis of these genome sequences, including synteny analyses, identification and classification of orthologous genes, and phylogenetic analysis was accomplished by the EDGAR software framework using default parameters [88]. In addition, the Artemis Comparison Tool (ACT) was applied to identify similarity between the different plasmids of *A. pasteurianus* 386B and *A. pasteurianus* IFO 3283 [89], using the BLASTN algorithm with default parameters [90].

### Data availability

The annotated sequences of the *A. pasteurianus* chromosome and plasmids were deposited in the DDBJ/EMBL/GenBank database (Sequencing Project PRJEB1172). The accession numbers are listed in Table 1.

## Abbreviations

AAB: Acetic acid bacteria; ACT: Artemis comparison tool; ADH: Alcohol dehydrogenase; AIMS: Architecture imparting sequences; ALDH: Aldehyde dehydrogenase; CDS: Protein-coding sequences; COG: Cluster of orthologous groups; CPSM: Cocoa pulp simulation medium; CRISPR: Clustered regularly interspaced short palindromic repeat; DHA: Dihydroxyacetone; EMP: Embden-Meyerhof-Parnas; FAD: Flavine adenine dinucleotide; GO: Gene ontology; LAB: Lactic acid bacteria; MYP: Mannitol-yeast extract-peptone; N5-CAIR: *N*^5^-carboxyaminoimidazole ribonucleotide; PPP: Pentose-phosphate pathway; PQQ: Pyrroloquinoline quinone; SCACT: Succinyl-CoA:acetate CoA transferase; TCA: Tricarboxylic acid.

## Competing interests

The authors declare that they have no competing interests.

## Authors’ contributions

KI carried out DNA extraction, conducted genome assembly and finishing, performed sequence annotation, bioinformatics analyses, and comparative genome analyses. SW and LDV designed and coordinated the study and participated in the analysis of the results. KI, SW, and LDV wrote the manuscript. All authors read and approved the final manuscript.

## Supplementary Material

Additional file 1 **Prophage- and transposon-related genes of *Acetobacter pasteurianus *386B.** Homologues of the genes involved are shown for *A. pasteurianus* IFO 3283, *Ga. diazotrophicus* Pal5*,* and *G. oxydans* 621H. Click here for file

Additional file 2 **Overview of genes in *Acetobacter pasteurianus *386B truncated by transposons in *A. pasteurianus *IFO 3283.** Overview of genomic positions of the homologues of truncated gene fragments of *A. pasteurianus* IFO 3283 in *A. pasteurianus* 386B. Click here for file

Additional file 3 **Strain-specific genes of *Acetobacter pasteurianus *386B.** Unique genes were identified by comparative analysis of five available genome sequences of *A. pasteurianus* strains using EDGAR. Click here for file

Additional file 4 **PCR assay for endopolygalacturonase gene.** Assays were performed on selected *A. pasteurianus* strains, originating from spontaneous cocoa bean fermentations. Click here for file

Additional file 5 **Uncharacterized oxidoreductases of *Acetobacter pasteurianus *386B.** Features of the genes that could be annotated are listed. The oxidoreductases are classified according to their protein family. Click here for file

Additional file 6 **Genes of *Acetobacter pasteurianus *386B coding for membrane-bound oxidoreductases with unknown specific function.** Homologues of the genes involved are shown for *A. pasteurianus* IFO 3283, *Ga. diazotrophicus* Pal5, and *G. oxydans* 621H. Click here for file

Additional file 7 **Genes of *Acetobacter pasteurianus *386B involved in thermotolerance.** List of homologous genes between *A. pasteurianus* 386B and *A. tropicalis* NBRC 101654, and between *A. pasteurianus* 386B and *A. pasteurianus* NBRC 101655. Click here for file
