# Supplementary material for: Complete genome sequence and comparative analysis of Acetobacter pasteurianus 386B, a strain well-adapted to the cocoa bean fermentation ecosystem
Source: BMC Genomics. 2013 Aug 1;14:526. doi: 10.1186/1471-2164-14-526 (PMC3751514; doi:10.1186/1471-2164-14-526)
Supplement: Additional file 4 — PCR assay for endopolygalacturonase gene. Assays were performed on selected A. pasteurianus strains, originating from spontaneous cocoa bean fermentations. [file 1471-2164-14-526-S4.docx]

**PCR assay for endopolygalacturonase gene**

To investigate the distribution of an endopolygalacturonase gene amongst different *Acetobacter pasteurianus* strains, a PCR assay targeting this gene was performed. The strains included in the screening originated from spontaneous cocoa bean fermentations carried out in Ghana (*A. pasteurianus* 386B), Brazil (*A. pasteurianus* D197, *A. pasteurianus* D91, *A. pasteurianus* A341b, and *A. pasteurianus* A304a), Ecuador (*A. pasteurianus* D748, *A. pasteurianus* D663, *A. pasteurianus* A1128, and *A. pasteurianus* A1228), and Malaysia (*A. pasteurianus* D1381 and *A. pasteurianus* D1095) [1-3]. Furthermore, *A. pasteurianus* LMG 1262^T^, the species’ type strain originating from beer obtained from the BCCM/LMG Bacteria Collection (Ghent, Belgium), was included as well [4]. Strains were transferred from -80 °C stock cultures into mannitol-yeast extract-peptone (MYP) medium (30 ml) and incubated aerobically in a rotary shaker at 150 rpm (Certomat BS-1; Sartorius AG, Göttingen, Germany) at 30 °C for 24 h. Subsequently, the strains were propagated in 30 ml of a cocoa pulp simulation medium (CPSM) for AAB [5] at 30 °C for 12 h. DNA isolation was performed as decribed before [6]. Two primer sets were designed using a consensus sequence of the endopolygalacturonase gene of *A. pasteurianus* 386B, *A. tropicalis* NBRC 101654, and *G. oxydans* H24 (Table 1). PCR assays were performed using a DNA T3000 thermocycler (Biometra, Göttingen, Germany) in a final volume of 50 μL. Following amplification, PCR product sizes were verified using a 1.0-% (w/v) agarose gel. Screening for the endopolygalacturonase gene resulted in the absence of this gene for all strains tested, except for *A. pasteurianus* 386B, and this for both primer sets tested. This indicates that the presence of an endopolygalacturonase gene is not widespread amongst strains of *Acetobacter pasteurianus*.

**Table 1 Primer properties.**

| Name | Sequence | Melting temperature (°C) |
| --- | --- | --- |
| EPG1F | CATGATGGCCGCACCAATGA | 58.5 |
| EPG1R | CAGAAACAAGGGCTTCACCG | 56.5 |
| EPG2F | ACGCAGACCTCAGGTCCTTT | 58.6 |
| EPG2R | TTCGCCAAAGCGAGACTGTG | 58.1 |

**References**

1. Papalexandratou Z, Vrancken G, De Bruyne K, Vandamme P, De Vuyst L: **Spontaneous organic cocoa bean box fermentations in Brazil are characterized by a restricted species diversity of lactic acid bacteria and acetic acid bacteria.** *Food Microbiol* 2011, **28:**1326-1338.

2. Papalexandratou Z, Falony G, Romanens E, Jimenez JC, Amores F, Daniel H-M, De Vuyst L: **Species diversity, community dynamics, and metabolite kinetics of the microbiota associated with traditional Ecuadorian spontaneous cocoa bean fermentations.** *Appl Environ Microbiol* 2011, **77:**7698-7714.

3. Camu N, De Winter T, Verbrugghe K, Cleenwerck I, Vandamme P, Takrama JS, Vancanneyt M, De Vuyst L: **Dynamics and biodiversity of populations of lactic acid bacteria and acetic acid bacteria involved in spontaneous heap fermentation of cocoa beans in Ghana.** *Appl Environ Microbiol* 2007, **73:**1809-1824.

4. Cleenwerck I, Vandemeulebroecke K, Janssens D, Swings J: **Re-examination of the genus *Acetobacter*, with descriptions of *Acetobacter cerevisiae* sp. nov. and *Acetobacter malorum* sp. nov.** *Int J Syst Evol Microbiol* 2002, **52:**1551-1558.

5. Lefeber T, Janssens M, Camu N, De Vuyst L: **Kinetic analysis of strains of lactic acid bacteria and acetic acid bacteria in cocoa pulp simulation media toward development of a starter culture for cocoa bean fermentation.** *Appl Environ Microbiol* 2010, **76:**7708-7716.

6. Ravyts F, Barbuti S, Frustoli MA, Parolari G, Saccani G, de Vuyst L, Leroy F: **Competitiveness and antibacterial potential of bacteriocin-producing starter cultures in different types of fermented sausages.** *J Food Prot* 2008, **71:**1817-1827.
